# Lipoprotein(a) Levels and Major Adverse Cardiovascular Events in Alberta, Canada: A Retrospective Cohort Study

**DOI:** 10.1016/j.cjco.2026.03.015

**Published:** 2026-04-01

**Authors:** Glen J. Pearson, Phongsack Manivong, Tram Pham, Eileen Shaw, Patrick Leclerc, Jonathan Fortier, Nada Farhat, Melanie Kok, Todd J. Anderson

**Affiliations:** a Faculty of Medicine and Dentistry, University of Alberta, Edmonton, Alberta, Canada; b Medlior Health Outcomes Research Ltd., Calgary, Alberta, Canada; c Novartis Pharmaceuticals Canada Inc., Montreal, Quebec, Canada; d Libin Cardiovascular Institute, Cumming School of Medicine, University of Calgary, Calgary, Alberta, Canada

**Keywords:** real-world evidence, atherosclerotic cardiovascular disease, lipoprotein(a), major adverse cardiovascular events

## Abstract

**Background:**

Elevated lipoprotein(a) (Lp(a)) is a key contributor to residual cardiovascular risk in secondary prevention populations. However, Lp(a) testing rates remain low despite guideline recommendations, highlighting a gap in atherosclerotic cardiovascular disease (ASCVD) management. The purpose of this study was to characterize patients with Lp(a) testing and established ASCVD and quantify the risk of subsequent major adverse cardiovascular events (MACE).

**Methods:**

A retrospective cohort study was conducted using administrative health data from Alberta, Canada. Patients were indexed on their first ASCVD event between January 1, 2015 and September 30, 2022. Time-to-first MACE was assessed using Kaplan–Meier and Cox regression models, grouped by different Lp(a) thresholds. Multivariable models were adjusted for significant characteristics identified through univariable analysis.

**Results:**

The study cohort included 6891 patients with a mean age of 60.0 years (standard deviation: 12.2), 35.9% female; 5163 had an Lp(a) level ≤ 50 mg/dL (∼120 nmol/L), and 1728 had an Lp(a) level > 50 mg/dL. At 8 years post-index, the MACE-free probability (95% confidence interval [CI]) was 76.3% (73.7%-79.0%) for ≤ 50 mg/dL, 64.7% (58.9%-70.4%) for > 50 mg/dL, 60.7% (53.4%-68.0%) for > 70 mg/dL (∼168 nmol/L), and 57.1% (46.8%-67.3%) for > 90 mg/dL (∼216 nmol/L). The adjusted hazard ratio (95% CI) for Lp(a) > 50, > 70, and > 90 mg/dL (vs the corresponding reference category) was 1.49 (1.29-1.73), 1.56 (1.33-1.83), and 1.60 (1.33-1.93), respectively.

**Conclusions:**

Patients with elevated Lp(a) level and ASCVD had increased risk of MACE. These findings support broader, routine Lp(a) testing to enable earlier intervention and inform use of targeted Lp(a)-lowering therapies in Canada.

## Résumé

**Contexte:**

Un taux élevé de lipoprotéine (a) [Lp(a)] est un déterminant clé du risque cardiovasculaire résiduel chez les populations relevant de la prévention secondaire. Cependant, les taux de dépistage de la Lp(a) restent faibles malgré les recommandations des lignes directrices, ce qui met en évidence une lacune dans la prise en charge des maladies cardiovasculaires athéroscléreuses (MCVAS). L'objectif de cette étude était de caractériser les patients ayant eu un test de dépistage de la Lp(a) et présentant une MCVAS établie, et de quantifier le risque d'événements cardiovasculaires indésirables majeurs (ECIM) ultérieurs.

**Méthodologie:**

Une étude de cohorte rétrospective a été menée à partir de données administratives de santé provenant de l'Alberta, au Canada. Les patients ont été indexés sur la base de leur premier événement de MCVAS survenu entre le 1^er^ janvier 2015 et le 30 septembre 2022. Le délai avant le premier ECIM a été évalué à l'aide des modèles de Kaplan-Meier et de régression de Cox, en regroupant les patients selon différents seuils de Lp(a). Les modèles multivariés ont été ajustés en fonction des caractéristiques significatives identifiées par l'analyse univariée.

**Résultats:**

La cohorte de l'étude comprenait 6 891 patients d'un âge moyen de 60,0 ans (écart-type : 12,2) et 35,9 % de femmes; 5 163 présentaient un taux de Lp(a) ≤ 50 mg/dl (∼120 nmol/l), et 1 728 un taux de Lp(a) > 50 mg/dl. Huit ans après l'événement initial, la probabilité d'absence d'ECIM (intervalle de confiance [IC] à 95 %) était de 76,3 % (73,7-79,0 %) pour un taux ≤ 50 mg/dl, de 64,7 % (58,9-70,4 %) pour un taux > 50 mg/dl, de 60,7 % (53,4-68,0 %) pour un taux > 70 mg/dl (∼168 nmol/l) et de 57,1 % (46,8-67,3 %) pour un taux > 90 mg/dl (∼216 nmol/l). Le rapport de risque ajusté (IC à 95 %) pour une Lp(a) > 50, > 70 et > 90 mg/dl (par rapport à la catégorie de référence correspondante) était respectivement de 1,49 (1,29-1,73), 1,56 (1,33-1,83) et 1,60 (1,33-1,93).

**Conclusions:**

Les patients présentant un taux élevé de Lp(a) et une MCVAS avaient un risque accru d'ECIM. Ces résultats plaident en faveur d’un dépistage systématique et plus large de la Lp(a) afin de permettre une intervention plus précoce et d’orienter le recours à des traitements ciblés visant à réduire la Lp(a) au Canada.

Despite advancements in treatment and prevention, atherosclerotic cardiovascular disease (ASCVD) remains a leading cause of morbidity and mortality in Canada and worldwide.^1^^,^^2^ Traditional risk factors, such as increased low-density lipoprotein cholesterol (LDL-C), can be effectively managed through both pharmacologic and lifestyle interventions; however, substantial residual cardiovascular (CV) risk persists in individuals with ASCVD, highlighting the need to address additional risk factors.^3^

Increasing evidence identifies lipoprotein(a) (Lp(a)) as a causal, independent, and clinically significant contributor to residual CV risk in individuals with ASCVD.^3^, ^4^, ^5^, ^6^ Lp(a) contributes to residual risk through several mechanisms, including enhanced arterial wall accumulation of cholesterol and oxidized phospholipids, proinflammatory effects, and prothrombotic activity by interfering with fibrinolysis.^7^^,^^8^ Notably, Lp(a) concentrations are predominantly genetically determined, accounting for approximately 70% to more than 90% of interindividual variability, and they are not modified by diet, exercise, or other lifestyle interventions.^4^ Several observational cohort studies conducted in the US, the United Kingdom (UK), and Asia have consistently demonstrated that an elevated Lp(a) level is associated with CV events and major adverse cardiovascular events (MACE) in secondary prevention populations.^9^, ^10^, ^11^, ^12^, ^13^, ^14^

Given the established role of Lp(a) as a CV risk factor, multiple professional societies recommend Lp(a) testing to guide risk management.^8^^,^^15^, ^16^, ^17^, ^18^ The 2021 Canadian Cardiovascular Society (CCS) Dyslipidemia Guidelines recommend that Lp(a) testing be completed once in every adult’s lifetime as part of initial lipid screening to improve CV risk.^15^ Similarly, recommendations from the European Society of Cardiology and the European Atherosclerosis Society^8^^,^^16^ advise measuring Lp(a) levels at least once in all adults, to identify those with elevated risk. Despite these clear recommendations from several professional societies, routine Lp(a) testing remains underutilized in clinical practice, even among high-risk populations.^19^, ^20^, ^21^, ^22^, ^23^ This underutilization represents a significant gap in ASCVD risk assessment and highlights the need for wider adoption of Lp(a) testing to guide clinical decision-making.

International research has established an association between elevated Lp(a) levels and increased risk of CV events in individuals with ASCVD; however, data specific to the Canadian population are limited. Given that Lp(a) is a clinically important contributor to residual risk in high-risk patients, characterizing individuals with ASCVD and elevated Lp(a) is important, to better identify those at increased risk. In a previous study, we demonstrated that patients with elevated Lp(a) levels and a history of ASCVD had higher healthcare resource utilization, costs, and rates of MACE.^24^ This study expands on previous findings by focusing on patients in Alberta, Canada with Lp(a) testing and ASCVD, examining their demographic and clinical characteristics, and time to subsequent MACE, by Lp(a) levels.

## 2 Materials and Methods

### Study design and setting

We conducted a retrospective observational cohort study using administrative heath and laboratory data from Alberta, Canada. The study complies with the 1975 Declaration of Helsinki, and research ethics board approval was obtained from the Health Research Ethics Board of Alberta—Community Health Committee (HREBA.CHC-23-0011). A waiver of consent from patients was approved due to the deidentified and anonymized nature of the data.

### Data sources

Administrative health data were provided by Alberta Primary & Preventative Health Services, and laboratory data were provided by Alberta Precision Laboratories. Administrative health data included inpatient hospitalizations (discharge abstract database), ambulatory care (national ambulatory care reporting system), practitioner claims, diagnostic imaging, pharmaceutical information network, vital statistics—deaths, and the Alberta Population Registry (Supplemental Table S1). The data contained anonymized (scrambled) patient identifiers, which allowed for data linkage across datasets.

### Study population

The study population included patients with Lp(a) testing and an ASCVD event between January 1, 2015 and September 30, 2022, with follow-up through March 31, 2023. The index date was the date of the first ASCVD event in the case ascertainment period. ASCVD was identified from inpatient hospitalizations, ambulatory care visits, and practitioner claims, using *International Classification of Diseases, 9th Revision, Clinical Modification* (ICD-9-CM) and *International Statistical Classification of Diseases and Related Health Problems, 10*^*th*^ *Revision, Canada* (ICD-10-CA) codes (Supplemental Table S2). Patients were excluded if they were non-Alberta residents or under 18 years of age at index, had less than 2 years of available lookback data prior to index, had a prior ASCVD event as early as January 1, 2013 (within 2-10 years) before the ASCVD index event, or died at the time of their ASCVD event.

### Variables

Demographic characteristics were measured at index and included sex, age, and Alberta Health Services geographic zone. To determine the last healthcare professional who was most likely responsible for diagnosing or overseeing the patient’s care, we identified the most recent provider visit within the 7 days prior to and including the index date. The provider categories were not mutually exclusive, as patients might have seen multiple providers or specialists on the same day. For Lp(a), the first instance of the test was captured, which may be before or after the ASCVD event. For other laboratory measurements, the last test performed within the 1 year prior to and including the index date was captured and included total cholesterol, LDL-C, high-density lipoprotein cholesterol, triglycerides, apolipoprotein B, and estimated glomerular filtration rate. Health conditions were identified for the 2 years prior to the index date and were defined using ICD-9-CM and ICD-10-CA codes from hospitalizations, ambulatory visits, and practitioner claims. These conditions included atrial fibrillation, hypertension, diabetes, heart failure, hypercholesterolemia, and dyslipidemia (hyperlipidemia) (Supplemental Table S3). Lastly, medication dispensations in the 1 year prior to index were described for statins by intensity (Supplemental Table S4), proprotein convertase subtilisin/kexin type 9 inhibitors, ezetimibe, angiotensin-converting enzyme inhibitors/angiotensin II receptor blockers, beta-blockers, calcium-channel blockers, antiplatelets, anticoagulants, fibrates, bile acid sequestrants, nicotinic acid and derivatives, and other lipid-modifying agents (Supplemental Table S5).

For the primary study outcome, a 4-point MACE composite was used, which included nonfatal myocardial infarction, nonfatal ischemic stroke, urgent coronary revascularization, and CV death. Each condition was defined using ICD-10-CA and Canadian Classification of Health Interventions codes from inpatient hospitalizations and the Alberta Vital Statistics death data (Supplemental Tables S6-S8). For the time-to-first MACE analyses, the first post-index MACE date was defined as the earlier of the admission date from the discharge abstract database or the death date from the Alberta Vital Statistics database. All results were stratified by Lp(a) levels ≤ 50 mg/dL (∼120 nmol/L) and > 50 mg/dL, as applicable, according to the thresholds outlined in the 2021 CCS Dyslipidemia Guidelines.^8^ Additional subgroups examined included Lp(a) levels of > 70 mg/dL and > 90 mg/dL, which correspond with the inclusion thresholds for novel Lp(a) targeted therapies.^25^^,^^26^

### Statistical analysis

All statistical analyses were conducted in SAS 9.4 (SAS Institute, Cary, NC). Baseline characteristics were analyzed descriptively, with categorical variables summarized using counts and percentages, and continuous variables summarized using means and standard deviations (SDs), or medians and interquartile ranges (IQRs). Kaplan–Meier and Cox proportional hazards models were used to perform the time-to-first MACE analyses following the index ASCVD event.

For the Kaplan–Meier analysis stratified by Lp(a) threshold ≤ 50 mg/dL and > 50 mg/dL, 3 χ^2^ tests of equality were performed that included log-rank, Wilcoxon (Breslow–Gehan–Wilcoxon), and likelihood ratio tests. Crude survival curves and probabilities at 6-month intervals were derived from Kaplan–Meier analysis for Lp(a) thresholds ≤ 50 mg/dL, > 50 mg/dL, > 70 mg/dL, and > 90 mg/dL.

For the Cox proportional hazards modelling, all baseline characteristics were considered as potentially relevant covariates in univariable models. A final multivariable adjusted Cox proportional hazards model that included 13 statistically significant variables from univariable analyses was performed separately using 3 Lp(a) thresholds (> 50, > 70, and > 90 mg/dL [∼120, 168, and 216 nmol/L, respectively]), with values at or below each threshold serving as the reference group. Direct adjusted survival curves (based on values of covariates in the cohort) and hazard ratios (HRs) for the Lp(a) thresholds and their accompanying 95% confidence intervals (CIs) were derived from the final Cox proportional hazards model.

Due to a lag in vital statistics data for mortality outcomes (cause of death), a sensitivity analysis was performed by restricting the cohort to the index date up to September 30, 2020 and ending follow-up on March 31, 2021. This analysis effectively reduced the accrual period and maximum follow-up by 2 years.

## 3 Results

A total of 8192 patients with Lp(a) testing and an ASCVD event were identified from January 1, 2015 to September 30, 2022 (Supplemental Fig. S1). After applying all exclusion criteria, the final cohort included 6891 patients, of which 5163 had Lp(a) levels ≤ 50 mg/dL, and 1728 had Lp(a) levels > 50 mg/dL. Notably, a large proportion (47.9%) of patients with Lp(a) levels > 50 mg/dL had Lp(a) levels > 90 mg/dL. Most patients had coronary artery disease (ie, stable/unstable angina, acute myocardial infarction, coronary atherosclerosis/historical myocardial infarction, percutaneous coronary intervention, or coronary artery bypass graft surgery) as an index ASCVD event (92.2% for ≤ 50 mg/dL, and 92.1% for > 50 mg/dL; Table 1). The median follow-up time was 2.1 years (IQR: 1.1-4.6) and 1.9 years (IQR: 1.0-4.4), for Lp(a) levels ≤ 50 mg/dL and > 50 mg/dL, respectively.

**Table 1:** Baseline characteristics stratified by baseline lipoprotein a (Lp(a)) levels

Characteristic∗	All Lp(a)	Lp(a) ≤ 50 mg/dL	Lp(a) > 50 mg/dL
Sample size	6891 (100.0)	5163 (74.9)	1728 (25.1)
**Total follow-up years**			
Mean (SD)	2.9 (2.3)	2.9 (2.3)	2.8 (2.3)
Median (IQR)	2.0 (1.0–4.5)	2.1 (1.1–4.6)	1.9 (1.0–4.4)
**Index ASCVD**†			
CAD	6350 (92.1)	4758 (92.2)	1592 (92.1)
CeVD	431 (6.3)	329 (6.4)	102 (5.9)
PAD	127 (1.8)	88 (1.7)	39 (2.3)
**Lp(a) group, mg/dL**			
≤ 50	5163 (74.9)	5163 (100.0)	
51–70	474 (6.9)		474 (27.4)
71–90	426 (6.2)		426 (24.7)
≥ 91	828 (12.0)		828 (47.9)
**Age at index**			
Mean (SD)	60.0 (12.2)	59.6 (12.4)	61.1 (11.5)
Median (IQR)	61.0 (52.0–69.0)	60.0 (51.0–68.0)	62.0 (54.0–69.0)
**Sex**			
Female	2473 (35.9)	1865 (36.1)	608 (35.2)
Male	4418 (64.1)	3298 (63.9)	1120 (64.8)
**Comorbidities**			
Atrial fibrillation	83 (1.2)	72 (1.4)	11 (0.6)
Diabetes	1186 (17.2)	877 (17.0)	309 (17.9)
Heart failure	292 (4.2)	214 (4.1)	78 (4.5)
Hypertension	2852 (41.4)	2103 (40.7)	749 (43.3)
Hypercholesterolemia	1281 (18.6)	925 (17.9)	356 (20.6)
Dyslipidemia	2136 (31.0)	1543 (29.9)	593 (34.3)
**Last specialist seen within 7 days prior to index**‡			
Cardiology	3498 (50.8)	2546 (49.3)	952 (55.1)
General/family physicians	1969 (28.6)	1494 (28.9)	475 (27.5)
Internal medicine	1484 (21.5)	1188 (23.0)	296 (17.1)
Other specialist	2279 (33.1)	1684 (32.6)	595 (34.4)
No specialist	8 (0.1)	∗1–4	∗1–4
**Laboratory measures, median (IQR)**			
Total cholesterol	4.8 (4.0–5.7)	4.8 (3.9–5.7)	4.8 (4.0–5.7)
LDL-C	2.8 (2.1–3.6)	2.8 (2.1–3.5)	2.8 (2.1–3.7)
HDL-C	1.2 (1.0–1.5)	1.2 (1.0–1.5)	1.2 (1.0–1.5)
Triglycerides	1.5 (1.0–2.3)	1.5 (1.0–2.3)	1.4 (1.0–2.2)
eGFR	82.0 (70.0–94.0)	82.0 (70.0–94.0)	82.0 (69.0–94.0)
Apolipoprotein B	0.9 (0.7–1.1)	0.9 (0.7–1.1)	0.9 (0.7–1.2)
**Statin intensity**			
(1) Low	121 (1.8)	92 (1.8)	29 (1.7)
(2) Moderate	1452 (21.1)	1071 (20.7)	381 (22.0)
(3) High	1658 (24.1)	1114 (21.6)	544 (31.5)
None	3660 (53.1)	2886 (55.9)	774 (44.8)
**Other baseline medications**			
PCSK9i	22 (0.3)	15 (0.3)	7 (0.4)
Ezetimibe	342 (5.0)	226 (4.4)	116 (6.7)

Values are n (%), unless otherwise indicated.

ASCVD, atherosclerotic cardiovascular disease; CAD, coronary artery disease; CeVD, cerebrovascular disease; eGFR, estimated glomerular filtration rate; HDL-C, high-density lipoprotein cholesterol; IQR, interquartile range; LDL-C, low-density lipoprotein cholesterol; Lp(a), lipoprotein(a); PAD, peripheral arterial disease; PCSK9i, proprotein convertase subtilisin/kexin type 9 inhibitor; SD, standard deviation.

∗ Percentages are with respect to column totals; values will sum to 100% within a column for characteristics where all categories are reported (Lp(a) group, sex, and statin intensity). Index ASCVD does not have mutually exclusive categories, as in rare instances, patients experience multiple events.

† CAD was defined as the following conditions using diagnostic codes in Supplemental Table S2: stable/unstable angina, acute myocardial infarction, coronary atherosclerosis/historical myocardial infarction, percutaneous coronary intervention, and coronary artery bypass graft surgery. CeVD was defined as the following conditions using diagnostic codes in Supplemental Table S2: stroke (ischemic) and transient ischemic attack.

‡ Some individuals see multiple specialists on the same day prior to their index date. Thus, percentages for the last seen specialist will not sum to 100%. "No specialist" indicates that the patient did not see a practitioner within 7 days prior to their index ASCVD.

### Baseline characteristics

Baseline characteristics of the overall study cohort and by Lp(a) levels ≤ 50 mg/dL and > 50 mg/dL are presented in Table 1 (additional characteristics are provided in Supplemental Table S9). Compared to individuals with Lp(a) levels ≤ 50 mg/dL at index, those with elevated Lp(a) levels (> 50 mg/dL) tended to be slightly older (mean [SD]: 61.1 [11.5] years vs 59.6 [12.4] years), with a numerically higher prevalence of comorbidities, including hypertension (43.3% vs 40.7%), hypercholesterolemia (20.6% vs 17.9%), and dyslipidemia (34.3% vs 29.9%). Patients with elevated Lp(a) levels also tended to be prescribed high-intensity statins at numerically higher rates, compared to those with lower levels (31.5% vs 21.6%).

### Time to first MACE

In the study cohort, 820 patients (11.9%) experienced a MACE during the follow-up period following their index ASCVD event. The distribution of the first MACE type following the index ASCVD event is shown in Supplemental Table S10, in which the most common events were myocardial infarction and coronary revascularization, regardless of Lp(a) level. At 8 years (96 months) of follow-up, the Kaplan–Meier MACE-free probability was 76.3% (95% CI: 73.7%-79.0%) for ≤ 50 mg/dL, 64.7% (95% CI: 58.9%-70.4%) for > 50 mg/dL, 60.7% (95% CI: 53.4%-68.0%) for > 70 mg/dL, and 57.1% (95% CI: 46.8%-67.3%) for > 90 mg/dL (Supplemental Table S11; Supplemental Fig. S2). All 3 χ^2^ tests of equality for the Lp(a) 50 mg/dL stratifications were statistically significant (*P* < 0.0001).

**Figure 1:**
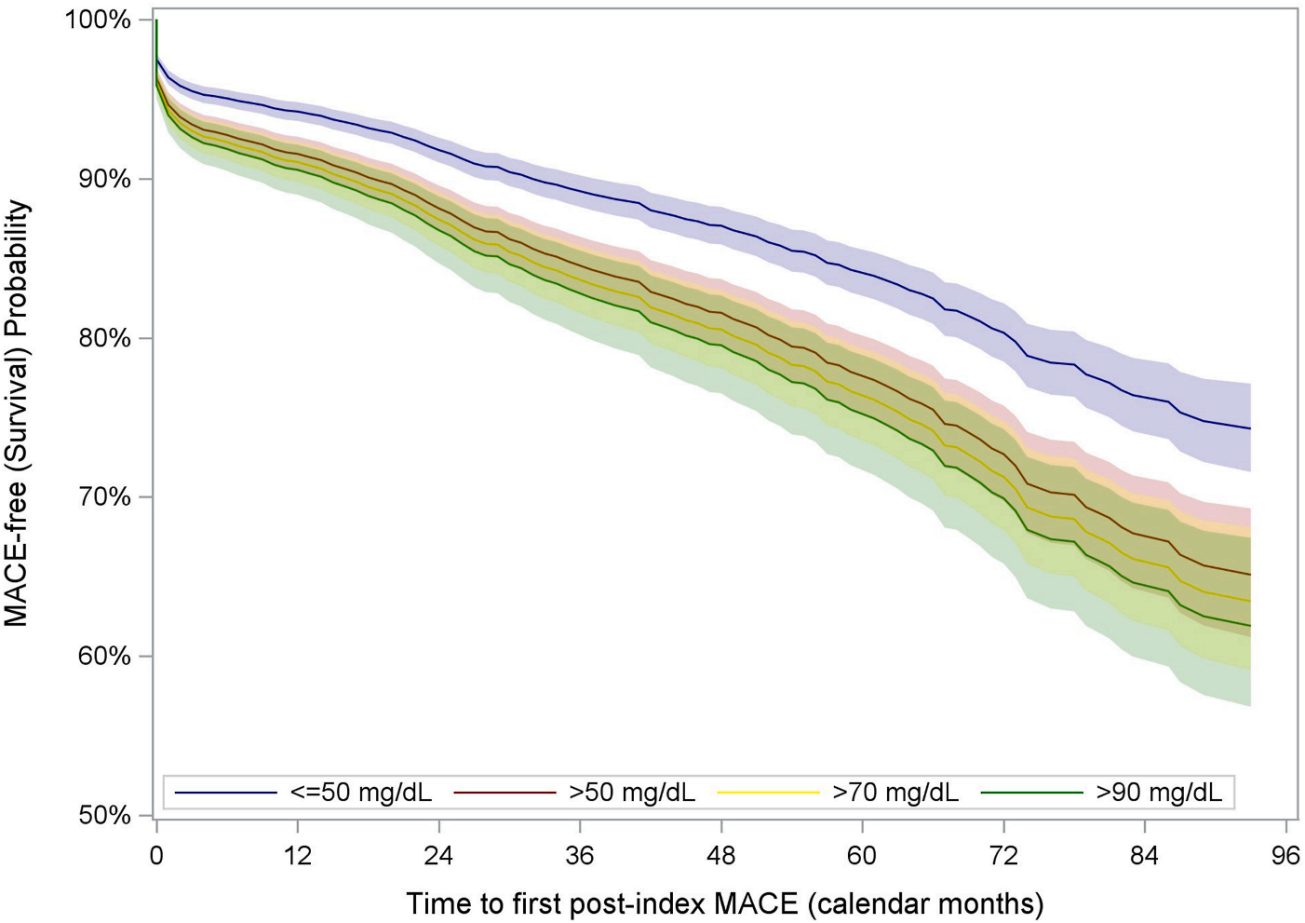
Adjusted (Cox proportional hazards model) survival probability (lipoprotein(a): ≤ 50 mg/dL, > 50 mg/dL, > 70 mg/dL, > 90 mg/dL). MACE, major adverse cardiovascular events.

**Figure 2:**
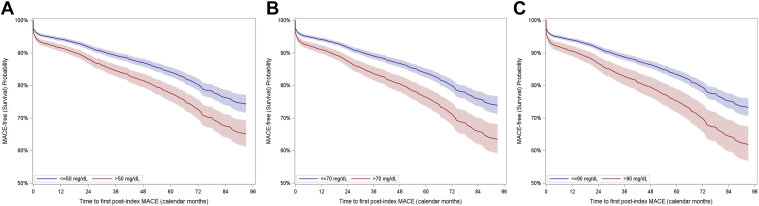
Adjusted (Cox proportional hazards model) survival probability (**A**) ≤ 50 mg/dL, > 50 mg/dL; (**B**) ≤ 70 mg/dL, > 70 mg/dL; (**C**) ≤ 90 mg/dL, > 90 mg/dL. MACE, major adverse cardiovascular events.

The HR and corresponding 95% CI for the 3 Lp(a) thresholds are presented in Table 2, and the adjusted survival probabilities are shown in Figure 1**,** and individually in Figure 2. The adjusted HR for Lp(a) > 50 mg/dL was 1.49 (95% CI: 1.29-1.73) relative to ≤ 50 mg/dL. The adjusted HR was numerically higher for Lp(a) levels > 70 mg/dL relative to ≤ 70 mg/dL (1.56, 95% CI: 1.33-1.83) and for Lp(a) levels > 90 mg/dL relative to ≤ 90 mg/dL (1.60, 95% CI: 1.33-1.93). Results of the sensitivity analyses restricting accrual end to September 30, 2020 and ending follow-up on March 31, 2021 demonstrated an even higher risk of MACE for those with elevated Lp(a), compared to the main analysis across all thresholds (Table 2).

**Table 2:** Cox proportional hazards time-to-first major adverse cardiovascular events (MACE) results

Lp(a) stratification, mg/dL	Univariate				Risk-adjusted				Sensitivity Analysis			
	HR	95% HR LCL	95% HR UCL	*P*	HR	95% HR LCL	95% HR UCL	*P*	HR	95% HR LCL	95% HR UCL	*P*
≤ 50	Ref	–	–	–	Ref	–	–	–	Ref	–	–	–
> 50	1.59	1.37	1.84	< 0.001	1.49	1.29	1.73	< 0.001	1.91	1.45	2.52	< 0.001
≤ 70	Ref	–	–	–	Ref	–	–	–	Ref	–	–	–
> 70	1.71	1.46	2.00	< 0.001	1.56	1.33	1.83	< 0.001	2.08	1.55	2.79	< 0.001
≤ 90	Ref	–	–	–	Ref	–	–	–	Ref	–	–	–
> 90	1.71	1.43	2.06	< 0.001	1.60	1.33	1.93	< 0.001	2.39	1.71	3.34	< 0.001

In the univariate analysis, 21 baseline covariates were examined, of which 13 were statistically significant (*P* < 0.05). In the adjusted model for the 50 mg/dL threshold, 6 variables remained significant: age, sex, Alberta Health Services geographic zone, estimated glomerular filtration rate, apolipoprotein B, and diabetes. For the 70 mg/dL and 90 mg/dL thresholds, the same variables, with the addition of hypertension, were statistically significant.

HR, hazard ratio; Lp(a), lipoprotein(a); MACE, major adverse cardiovascular events; LCL, lower confidence limit; Ref, reference category; UCL, upper confidence limit.

A dash indicates that the value is not applicable for the reference group.

## 4 Discussion

This study provides valuable insights into the characteristics and time to MACE among a secondary prevention population of patients with Lp(a) testing and ASCVD in Alberta, Canada. Among patients with ASCVD, those with elevated Lp(a) levels were generally older, had more comorbidities, and were often prescribed high-intensity statins, compared to those with lower Lp(a) levels. However, they remained at higher risk of MACE, highlighting that residual CV risk is not fully mitigated by conventional lipid-lowering therapies. The findings offer population-based Canadian-specific data, and unlike most prior studies, our analysis utilized a time-to-event analytical approach across 3 distinct, clinically relevant Lp(a) threshold levels, offering a more nuanced understanding of risk stratification.

Population-based estimates from Alberta indicate a substantial burden of ASCVD. Between 2012 and 2016, a total of 198,573 patients were identified with clinical ASCVD, corresponding to a 5-year period prevalence of 89.9 per 1000 persons (approximately 9%).^27^ Within our study cohort, patients with ASCVD represented more than one-quarter of all patients who underwent Lp(a) testing (Supplemental Fig. S1**)**, further highlighting the clinical relevance of this population.

Our results contribute to the growing body of evidence demonstrating that elevated Lp(a) increases the risk of CV events and MACE in secondary prevention populations.^9^, ^10^, ^11^, ^12^, ^13^, ^14^ These findings are comparable to recent real-world analyses from the US and UK examining patients with ASCVD.^9^^,^^10^ The large retrospective US cohort study using claims data reported a 45% (HR: 1.45 [95% CI:1.39-1.51]) increased risk of recurrent ASCVD events, after adjusting for covariates, among individuals with ASCVD and Lp(a) levels ≥ 300 nmol/L (approximately 125 mg/dL).^9^ In contrast, our study demonstrated a 60% increased risk of MACE for those with Lp(a) > 90 mg/dL in an adjusted model, indicating a similar but slightly stronger association. Additionally, the UK study reported an 8% increased risk of MACE (HR: 1.08 [95% CI: 1.06-1.10]) per 100 nmol/L (∼42 mg/dL) increase in Lp(a), indicating a modest incremental risk increase.^10^ In contrast, our study, using discrete mg/dL thresholds, found HRs ranging from 1.49 to 1.60 for progressively higher Lp(a) levels, suggesting a stronger association.

Although Lp(a) level is established as a causal and independent risk factor for ASCVD, it remains underutilized in clinical practice. This underuse is driven by multiple factors, including the perception that Lp(a) testing has limited practical impact on clinical management, given the lack of Lp(a)-specific therapies, as well as low clinician awareness and familiarity.^19^, ^20^, ^21^, ^22^, ^23^ Consequently, Lp(a) testing is often reserved for select high-risk patients or specialty settings rather than incorporated as a routine clinical measurement.

Additionally, recommendations for Lp(a) testing by professional societies remain inconsistent. The CCS, European Society of Cardiology/European Atherosclerosis Society, and National Lipid Association support at least one lifetime measurement of Lp(a),^15^^,^^16^^,^^18^ particularly in high-risk individuals, but do not universally recommend repeated or routine Lp(a) testing as part of standard lipid panels. Additionally, the PEER (Patients Experience Evidence Research) Simplified Lipid Guideline does not support routine Lp(a) measurement, citing the absence of approved Lp(a)-specific therapies and limited evidence that testing leads to meaningful changes in patient outcomes.^28^ Nevertheless, evidence suggests that Lp(a) testing can influence clinical management. A retrospective study of 5 large US health systems found that patients who underwent Lp(a) testing were more likely to initiate a lipid-lowering therapy, regardless of Lp(a) levels.^20^ In addition, among those with an elevated Lp(a) level, clinicians were more likely to initiate intensive therapies, such as proprotein convertase subtilisin/kexin type 9 inhibitors and ezetimibe, highlighting the potential of Lp(a) testing to guide more aggressive risk reduction strategies in clinical practice.^20^

Currently, no specific therapies are approved for lowering Lp(a) level, although novel treatments are under investigation. Single-stranded antisense oligonucleotides (ASO) and small interfering ribonucleic acid (siRNA) have been developed to specifically target apolipoprotein(a) messenger RNA to reduce Lp(a) concentrations.^29^^,^^30^ Notably, agents such as pelacarsen, olpasiran, lepodisiran, and SLN360, which utilize these RNA-targeted approaches, have demonstrated efficacy in lowering Lp(a) levels in clinical trials.^29^, ^30^, ^31^

In the absence of approved therapies, Lp(a) testing remains important for identifying patients at elevated risk and guiding the intensification of standard risk-reduction strategies, including aggressive lowering of LDL-C level and optimization of other modifiable cardiovascular risk factors. This approach is consistent with current guideline recommendations, which emphasize comprehensive risk reduction rather than Lp(a)-directed treatment in patients with established ASCVD. However, as dedicated Lp(a)-lowering therapies become available, routine measurement will become increasingly critical for identifying patients who may benefit from targeted treatment.

### Strengths and Limitations

A major strength of this study was the use of a large set of population-based administrative health data, along with province-wide laboratory data, which enabled a comprehensive assessment of Lp(a) testing in Alberta. Additionally, the long study follow-up time available (up to 8 years [96 months]) allowed for a comprehensive assessment of MACE over time at the population level.

These findings should also be interpreted with caution, as administrative data are not collected specifically for research purposes and are subject to inherent limitations. Clinical characteristics, such as comorbidities, may be inaccurately recorded due to coding errors or underreporting. Additionally, laboratory data coverage was incomplete across the province for the entire study period. Specifically, data from Edmonton were unavailable prior to January 2016 and were partially missing from January 2020 to December 2023. Finally, the requirement for Lp(a) testing to be included in the cohort may have resulted in a higher-risk population, although this aligns with current clinical testing guidelines recommending Lp(a) measurement in patients with established ASCVD and suspected elevated risk.

## Conclusion

Our population-based Canadian study demonstrates that elevated Lp(a) levels are associated with a higher risk of MACE among patients with ASCVD, highlighting its importance for risk stratification and identifying patients at increased risk of subsequent events, who may benefit from more intensive management. In line with clinical guidelines that recommend at least once-in-lifetime Lp(a) measurement, our findings provide real-world evidence supporting the need for greater awareness and systematic uptake of Lp(a) testing. These findings strongly support the integration of broader and routine Lp(a) testing into secondary prevention strategies, to enable earlier clinical intervention and inform the future use of targeted Lp(a)-lowering therapies.

## Disclaimer

This study is based on data provided by Primary & Preventative Health Services (PPHS) and Alberta Precision Laboratories. The interpretation and conclusions contained herein are those of the researchers and do not necessarily represent the views of the Government of Alberta. Neither the Government of Alberta nor PPHS express any opinion in relation to this study.

## Acknowledgements

The authors thank Cristiano Soares de Moura for his support in data validation for our study as an employee of Medlior, which received funding from Novartis Pharmaceuticals Canada, Inc.

## Data Availability

De-identified data were released to Medlior Health Outcomes Research by PPHS following ethical approval and a data request. Data from this study are not publicly available and cannot be shared due to privacy reasons and ethical restrictions, per the research agreement with PPHS and Alberta Precision Laboratories.

## Ethics Statement

Research ethics board approval was obtained from the Health Research Ethics Board of Alberta—Community Health Committee (HREBA.CHC-23-0011).

## Declaration of Generative AI and AI-Assisted Technologies

During the preparation of this work, the authors used ChatGPT for copy editing purposes. After using this tool, the authors reviewed and revised the content as needed and take full responsibility for the content of the published article.

## Patient Consent

The authors confirm that patient consent is not applicable to this article, as a waiver of consent was approved by the Health Research Ethics Board of Alberta—Community Health Committee (HREBA.CHC-23-0011) due to the deidentified and anonymized nature of the data.

## Footnotes

To access the supplementary material accompanying this article, visit *CJC Open* at https://www.cjcopen.ca/ and at https://doi.org/10.1016/j.cjco.2026.03.015
