# Supplementary material for: Lipoprotein(a) Levels and Major Adverse Cardiovascular Events in Alberta, Canada: A Retrospective Cohort Study
Source: CJC Open. 2026 Apr 1;8(8):981–8. doi: 10.1016/j.cjco.2026.03.015 (PMC13480158; doi:10.1016/j.cjco.2026.03.015)
Supplement: Supplementary file 1 [file mmc1.pdf]

Supplemental Table S1 Data Sources

Dataset	Source	Coverage Period
Alberta Blue Cross Pharmacy Claims	Alberta Health	January 1, 2013 to March 31, 2023
Ambulatory Care – National Ambulatory Care Reporting System	Alberta Health	January 1, 2005 to March 31, 2023
Inpatient Hospitalizations – Discharge Abstract Database	Alberta Health	January 1, 2005 to March 31, 2023
Laboratory Data	Alberta Precision Laboratories	January 1, 2013 to March 31, 2023
Diagnostic Imaging	Alberta Health	January 1, 2013 to March 31, 2023
Pharmaceutical Information Network Dispenses	Alberta Health	January 1, 2013 to March 31, 2023
Alberta Population Registry	Alberta Health	January 1, 2013 to March 31, 2023
Practitioner Claims	Alberta Health	January 1, 2005 to March 31, 2023
Vital Statistics – Deaths	Alberta Health	January 1, 2015 to March 31, 2023

Supplemental Table S2 ASCVD ICD-9-CM and ICD-10-CA definition

ASCVD Conditions	ICD-9-CM	ICD-10-CA	Definition Algorithm[a-f]
Acute myocardial infarction	410	I21, I22	• 1 inpatient hospitalization<br>• Any position
Stable/unstable angina	411, 413	I20	• 1 inpatient hospitalization or 1 physician claim or 1 ED visit<br>• Any position
Ischemic stroke	434, 436	I63 (excluding I63.6), I64	• 1 inpatient hospitalization or 2 physician claims/ED visits at least 30 days apart<br>• Any position
Transient ischemic attack	435	G45.0-G45.3, G45.8-G45.9, H34.0	• 1 inpatient hospitalization or 2 physician claims/ED visits at least 30 days apart<br>• Any position
Coronary atherosclerosis/ historical myocardial infarction	412, 414	I25	• 1 inpatient hospitalization or 2 physician claims/ED visits at least 30 days apart<br>• Any position
Peripheral arterial disease	443.9, 440.2	I73.9, I70.2, I79.2	• 1 inpatient hospitalization or 2 physician claims/ED visits at least 30 days apart<br>• Any position
Percutaneous coronary intervention	CCP codes: 4802, 4803	CCI codes: 1IJ50, 1IJ57GQ, 1IJ54	• 1 inpatient hospitalization or 1 ambulatory care visit<br>• Any position
Coronary artery bypass graft surgery	CCP codes: 481	CCI codes: 1IJ76	• 1 inpatient hospitalization or 1 ambulatory care visit<br>• Any position

Abbreviations: CCI: Canadian Classification of Health Interventions; CCP: Canadian Classification of Diagnostic, Therapeutic, and Surgical Procedures; ED: emergency department; ICD 9-CM: The International Classification of Diseases, Ninth Revision, Clinical Modification; ICD-10-CA: International Statistical Classification of Diseases and Related Health Problems, 10th Revision, Canada

Note: Modified from Recent Temporal Changes in Atherosclerotic Cardiovascular Diseases in Ontario: Clinical and Health Systems Impact Tu, Jack V. et al. Canadian Journal of Cardiology, Volume 33, Issue 3, 378 – 384
http://dx.doi.org/10.1016/j.cjca.2016.11.009

[a] For conditions involving inpatient hospitalizations, the event date is procedure date if applicable and available, otherwise, it is discharge date.

[b] For conditions involving claims, the event date is service date.

[c] For conditions involving ER, the event date is registration date.

[d] For conditions requiring 2 encounters, the event date is the second encounter.
[e] For each condition, the diagnosis or index date is the first (earliest) event.
[f] Any position refers to any diagnosis or intervention field in the Discharge Abstract Database (inpatient), National Ambulatory Care Reporting System (ED visits), or Practitioner Claims datasets.

Supplemental Table S3 Baseline Conditions

Clinical Characteristic	ICD-9-CM	ICD-10-CA	Definition / Algorithm[f,g]
Atrial fibrillation[a]	427.3	I48	• 2 healthcare encounters 30+ days apart but within 365 days from inpatient, ED visit or Claim visits<br>• 2 Claims visits <30 days apart on different days is considered 1 encounter<br>• Any position
Hypertension[b]	401-405	I10-I13, I15	• 1 inpatient or 2 Claims visits on different days within 3 years (1,095 days)<br>• Any position
Diabetes[c]	250	E10-E14	• 1 inpatient or 2 Claim visits within 2 years (730 days)<br>• Any position
Heart failure[d]	428.0, 428.1, 429.8	I09.9, I11.0, I13.0, I13.2, I25.5, I42.0, I42.5, I42.9, I43, I50, P29.0	• 1 inpatient or 1 Ambulatory visit<br>• Any position
Hypercholesterolemia[e]	272 (excluding 272.1 and 272.6)	E78.x (excluding E78.1, E78.71, E78.72, E78.81)	• 1 inpatient or 1 Claim visit<br>• Any position
Dyslipidemia (hyperlipidemia)	272	E78	• 1 inpatient, 1 ED visit or 1 Claim visit<br>• Any position

Abbreviations: ED: emergency department; ICD 9-CM: The International Classification of Diseases, Ninth Revision, Clinical Modification; ICD-10-CA: International Statistical Classification of Diseases and Related Health Problems, 10th Revision, Canada

[a] Modified from Alberta IHDA Chronic Disease – Atrial Fibrillation http://www.ahw.gov.ab.ca/IHDA_Retrieval/redirectToURL.do?cat=6&subCat=555

[b] Modified from Alberta IHDA Chronic Disease – Hypertension and Diabetes http://www.ahw.gov.ab.ca/IHDA_Retrieval/redirectToURL.do?cat=6&subCat=144

[c] Definition from Alberta IHDA Chronic Disease – Diabetes http://www.ahw.gov.ab.ca/IHDA_Retrieval/redirectToURL.do?cat=6&subCat=121

[d] Definition from Alberta IHDA Chronic Disease – Congestive Heart Failure http://www.ahw.gov.ab.ca/IHDA_Retrieval/redirectToURL.do?cat=6&subCat=789

[e] Definition from Chen, et al. "Prevalence of atherosclerotic cardiovascular disease and subsequent major adverse cardiovascular events in Alberta, Canada: A real-world evidence study." Clinical cardiology vol. 44,11 (2021): 1613-1620. doi:10.1002/clc.23732

[f] Any position refers to any diagnosis or intervention field in the Discharge Abstract Database (IP), National Ambulatory Care Reporting System (ED visits), or Practitioner Claims datasets.
[g] Diabetes and hypertension records for females aged 10 to 54 are excluded if the diagnosis occurs 150 days before or after a pregnancy-related visit (ICD-9: 641-6767 or V27, ICD-10: O1, O21-O95, O98, O99, and Z37).

Supplemental Table S4 List of ATC codes for statin treatment

ATC Code	Statin Drug Name	Low Intensity	Moderate Intensity	High Intensity
C10AA01	Simvastatin	10 mg	20-40 mg	80 mg
C10AA02	Lovastatin	10-20 mg	40 mg	
C10AA03	Pravastatin	10-20 mg	40-80 mg	
C10AA04	Fluvastatin	20-40 mg	40-80 mg	
C10AA05	Atorvastatin		10-20 mg	40-80 mg
C10AA07	Rosuvastatin		5-10 mg	20-40 mg
C10AA08	Pitavastatin	1 mg	2-4 mg	

Abbreviations: ATC: anatomical therapeutic chemical
Source: https://www.whocc.no/atc_ddd_index/?code=C10&showdescription=no

Supplemental Table S5 List of Other Medications[1]

Drug Class	ATC	Subgroup / Drug Name
Anticoagulant	B01AA	Vitamin K antagonists
Anticoagulant	B01AB	Heparin group
Antiplatelet	B01AC	Platelet aggregation inhibitors excl. heparin
Anticoagulant	B01AD	Enzymes
Anticoagulant	B01AE	Direct thrombin inhibitors
Anticoagulant	B01AF	Direct factor Xa inhibitors
Other Antithrombotic	B01AX	Other antithrombotic agents
Beta Blocker	C07A	Beta Blocking Agents
Beta Blocker	C07B	Beta Blocking Agents and Thiazides
Beta Blocker	C07C	Beta Blocking Agents and Other Diuretics
Beta Blocker	C07D	Beta Blocking Agents, Thiazides and Other Diuretics
Beta Blocker	C07E	Beta Blocking Agents and Vasodilators
Beta Blocker	C07F	Beta Blocking Agents, Other Combinations
CCB	C08CA	Dihydropyridine derivatives
CCB	C08CX	Other selective CCBs with mainly vascular effects
CCB	C08DA	Phenylalkylamine derivatives
CCB	C08DB	Benzothiazepine derivatives
CCB	C08EA	Phenylalkylamine derivatives
CCB	C08EX	Other non-selective calcium channel blockers
CCB	C08GA	Calcium channel blockers and diuretics
ACEi	C09AA	ACE inhibitors, plain
ACEi	C09BA	ACE inhibitors and diuretics
ACEi	C09BB	ACE inhibitors and calcium channel blockers
ACEi	C09BX	ACE inhibitors, other combinations
ARB	C09CA	Angiotensin II receptor blockers (ARBs), plain
ARB	C09DA	Angiotensin II receptor blockers (ARBs) and diuretics
ARB	C09DB	Angiotensin II receptor blockers (ARBs) and CCBs
ARB	C09DX	Angiotensin II receptor blockers (ARBs), other combinations
Lipid Modifying Agent	C10AB	Fibrates
Lipid Modifying Agent	C10AC	Bile acid sequestrants
Lipid Modifying Agent	C10AD	Nicotinic acid and derivatives
Lipid Modifying Agent	C10AX06	Omega-3-Triglycerids Included Other Esters & Acids
Lipid Modifying Agent	C10AX09	Ezetimibe
Lipid Modifying Agent	C10AX12	Lomitapide

Drug Class	ATC	Subgroup / Drug Name
Lipid Modifying Agent	C10AX13	PCSK9i-Evolocumab
Lipid Modifying Agent	C10AX14	PCSK9i-Alirocumab

Abbreviations: ACEi: angiotensin-converting enzyme inhibitor; ARB; angiotensin II receptor blocker; ATC: anatomical therapeutic chemical; CCB: calcium channel blockers; PCSK9i: Proprotein convertase subtilisin/kexin type 9 inhibitor

[1]https://health-products.canada.ca/dpd-bdpp/index-eng.jsp

Supplemental Table S6 Major Adverse Cardiovascular Events (MACE)

MACE Outcomes (Parameter)[a]	ICD-10-CA	CCI	Definition / Algorithm[b,c]
Non-fatal myocardial infarction	I21, I22		• 1 inpatient hospitalization<br>• Any position (excluding Type 1 for pre-admit comorbidity)<br>• A final visit/discharge disposition that is not death
Non-fatal ischemic stroke	I63 (excluding I63.6), I64		• 1 inpatient hospitalization<br>• Any position (excluding Type 1 for pre-admit comorbidity)<br>• A final visit/discharge disposition that is not death
Urgent coronary revascularization		1IJ50, 1IJ57GQ, 1IJ54, 1IJ76	• 1 inpatient hospitalization<br>• Any position<br>• An admission category of "U" (Urgent/Emergent)
Cardiovascular death[d]	I00-I99		• Vital Statistics record for a death or 1 inpatient hospitalization with discharge disposition of death<br>• Any position
MACE (Composite)			The occurrence of any of the following events: non-fatal myocardial infarction, non-fatal stroke, urgent coronary revascularization, and cardiovascular death

Abbreviations: CCI: Canadian Classification of Health Interventions; ICD-10-CA: International Statistical Classification of Diseases and Related Health Problems, 10th Revision, Canada; MACE: major adverse cardiovascular events

[a] MACE sub-components may not be mutually exclusive (i.e., the sum of individual MACE components may not sum to the composite MACE).

[b] For the MACE count/rate analysis, all MACE events will be included, and not just the first event. A washout period of 30 days (from discharge to the next admission) will be used to identify new events.

[c] See **Supplemental Table S7** for the full list of diagnosis types in DAD.

[d] See **Supplemental Table S8** for the list of disposition codes related to death.

Source: Codes definition from Medlior-AMGEN study "ASCVD, Health Outcomes and Healthcare Resource Utilization (HCRU) in Alberta: A Real-world Evidence Study"

Publication: Chen, et al. "Prevalence of atherosclerotic cardiovascular disease and subsequent major adverse cardiovascular events in Alberta, Canada: A real-world evidence study." Clinical cardiology vol. 44,11 (2021): 1613-1620. doi:10.1002/clc.23732

Supplemental Table S7 Diagnosis Type in Discharge Abstract Database

Code	Type Description
M	Most responsible diagnosis
1	Comorbidity-preadmit
2	Comorbidity-post admit
3	Secondary diagnosis
5	Admitting diagnosis
6	Proxy most responsible diagnosis
W, X, Y	Service transfer diagnosis
9	External cause of injury codes
0	Newborn discharge abstracts

Source: Canadian Institute for Health Information. DAD Abstracting Manual, 2022–2023. Ottawa, ON: CIHI; 2022.

Supplemental Table S8 Death-Related Disposition Codes[a]

Applicable Fiscal Years	Disposition Code	Description
2014/15-2017/18	07	Died (Discharge Abstract Database only)
	10	Death after Arrival (NACRS only)
	11	Death on Arrival (NACRS only)
2018/19-Present	71	Dead on Arrival
	72	Died in Facility

Abbreviations: NACRS; National Ambulatory Care Reporting System
[a]Excludes suicide and medical assistance in dying.

Sources:

[1]Canadian Institute for Health Information. DAD Abstracting Manual, 2016–2017. Ottawa, ON: CIHI; 2016
[2]Canadian Institute for Health Information. DAD Abstracting Manual, 2022–2023. Ottawa, ON: CIHI; 2022
[3]Canadian Institute for Health Information. National Ambulatory Care Reporting System Abstracting Manual, 2016–2017. Ottawa, ON: CIHI; 2016
[4]Canadian Institute for Health Information. National Ambulatory Care Reporting System Abstracting Manual, 2022–2023. Ottawa, ON: CIHI; 2022

Supplemental Table S9 Additional baseline characteristics of the study cohort, stratified by baseline Lp(a) levels

Characteristic	All Lp(a)	<=50 mg/dL	>50 mg/dL
Sample size (n)	6,891 (100.0%)	5,163 (74.9%)	1,728 (25.1%)
**Age Group (Years), n (%)**			
18-44 years	786 (11.4%)	633 (12.3%)	153 (8.9%)
45-54 years	1,362 (19.8%)	1,050 (20.3%)	312 (18.1%)
55-64 years	2,121 (30.8%)	1,562 (30.3%)	559 (32.3%)
65-74 years	1,827 (26.5%)	1,335 (25.9%)	492 (28.5%)
75+ years	795 (11.5%)	583 (11.3%)	212 (12.3%)
**AHS Geographic Zone, n (%)**			
Z1-South	658 (9.5%)	501 (9.7%)	157 (9.1%)
Z2-Calgary	4,434 (64.3%)	3,211 (62.2%)	1,223 (70.8%)
Z3-Central	268 (3.9%)	215 (4.2%)	53 (3.1%)
Z4-Edmonton	1,255 (18.2%)	1,008 (19.5%)	247 (14.3%)
Z5-North	275 (4.0%)	227 (4.4%)	48 (2.8%)
Missing	*1-4	*1-4	
**Total Cholesterol, n (%)**			
<5.20 mmol/L	2,589 (37.6%)	1,916 (37.1%)	673 (38.9%)
5.20-6.29 mmol/L	1,012 (14.7%)	736 (14.3%)	276 (16.0%)
6.30+ mmol/L	517 (7.5%)	376 (7.3%)	141 (8.2%)
Unavailable	2,773 (40.2%)	2,135 (41.4%)	638 (36.9%)
**LDL-C, n (%)**			
<1.40 mmol/L	410 (5.9%)	318 (6.2%)	92 (5.3%)
1.40-1.79 mmol/L	465 (6.7%)	345 (6.7%)	120 (6.9%)
1.80-1.99 mmol/L	292 (4.2%)	208 (4.0%)	84 (4.9%)
2.00-2.59 mmol/L	982 (14.3%)	736 (14.3%)	246 (14.2%)
2.60-3.59 mmol/L	1,670 (24.2%)	1,246 (24.1%)	424 (24.5%)
3.60+ mmol/L	1,246 (18.1%)	896 (17.4%)	350 (20.3%)
Unavailable	1,826 (26.5%)	1,414 (27.4%)	412 (23.8%)
**HDL-C, n (%)**			
<1.00 mmol/L	1,443 (20.9%)	1,090 (21.1%)	353 (20.4%)
1.00-1.59 mmol/L	2,874 (41.7%)	2,113 (40.9%)	761 (44.0%)
1.60+ mmol/L	885 (12.8%)	656 (12.7%)	229 (13.3%)
Unavailable	1,689 (24.5%)	1,304 (25.3%)	385 (22.3%)
**Triglycerides, n (%)**			
<1.5 mmol/L	338 (4.9%)	264 (5.1%)	74 (4.3%)
1.50-2.29 mmol/L	163 (2.4%)	131 (2.5%)	32 (1.9%)
2.30-5.69 mmol/L	132 (1.9%)	106 (2.1%)	26 (1.5%)
5.70+ mmol/L	25 (0.4%)	22 (0.4%)	*1-4

**Characteristic**	**All Lp(a)**	**<=50 mg/dL**	**>50 mg/dL**
Unavailable	6,233 (90.5%)	4,640 (89.9%)	1,593 (92.2%)
**eGFR, n (%)**			
<30 mL/min/1.73m^2	58 (0.8%)	46 (0.9%)	12 (0.7%)
30-59 mL/min/1.73m^2	603 (8.8%)	446 (8.6%)	157 (9.1%)
60+ mL/min/1.73m^2	4,928 (71.5%)	3,639 (70.5%)	1,289 (74.6%)
Unavailable	1,302 (18.9%)	1,032 (20.0%)	270 (15.6%)
**Apolipoprotein B, n (%)**			
<0.70 g/L	168 (2.4%)	140 (2.7%)	28 (1.6%)
0.70-0.79 g/L	96 (1.4%)	72 (1.4%)	24 (1.4%)
0.80-1.04 g/L	241 (3.5%)	188 (3.6%)	53 (3.1%)
1.05-1.45 g/L	208 (3.0%)	161 (3.1%)	47 (2.7%)
1.46+ g/L	25 (0.4%)	18 (0.3%)	7 (0.4%)
Unavailable	6,153 (89.3%)	4,584 (88.8%)	1,569 (90.8%)
**Other Baseline Medications, n (%)**			
ACEi/ARBs	3,001 (43.5%)	2,214 (42.9%)	787 (45.5%)
Anticoagulants	469 (6.8%)	368 (7.1%)	101 (5.8%)
Antiplatelets	1,294 (18.8%)	901 (17.5%)	393 (22.7%)
Beta blockers	1,829 (26.5%)	1,343 (26.0%)	486 (28.1%)
Bile acid sequestrants	30 (0.4%)	21 (0.4%)	9 (0.5%)
Calcium channel blockers	1,059 (15.4%)	791 (15.3%)	268 (15.5%)
Fibrates	121 (1.8%)	98 (1.9%)	23 (1.3%)
Nicotinic acid and derivatives	*1-4	*1-4	*1-4
Other lipid modifying agents	0 (0.0%)	0 (0.0%)	0 (0.0%)

Abbreviations: ASCVD: atherosclerotic cardiovascular disease; CAD: coronary artery disease; CeVD: cerebrovascular disease; eGFR: estimated glomerular filtration rate; HDL-C: high-density lipoprotein cholesterol; IQR: interquartile range; LDL-C: low-density lipoprotein cholesterol; Lp(a): lipoprotein(a); PAD: peripheral arterial disease

[a] Percents are with respect to column totals, so values will sum to 100% within a column for characteristics where all categories are reported (all characteristics except baseline medications).

Supplemental Table S10 Distribution of First Post-ASCVD MACE Type

	Cohort						
	**MACE Patients**	**Lp(a)<=50 mg dL**	**Lp(a)>50 mg dL**	**Lp(a)<=70 mg dL**	**Lp(a)>70 mg dL**	**Lp(a)<=90 mg dL**	**Lp(a)>90 mg dL**
Sample size (n)	820	548	272	610	210	681	139
**MACE, n (%)[a]**							
Myocardial Infarction	471 (57.4%)	313 (57.1%)	158 (58.1%)	346 (56.7%)	125 (59.5%)	389 (57.1%)	82 (59.0%)
Ischemic Stroke	83 (10.1%)	62 (11.3%)	21 (7.7%)	67 (11.0%)	16 (7.6%)	74 (10.9%)	9 (6.5%)
Coronary Revascularization	555 (67.7%)	359 (65.5%)	196 (72.1%)	400 (65.6%)	155 (73.8%)	449 (65.9%)	106 (76.3%)
Cardiovascular Death	41 (5.0%)	34 (6.2%)	7 (2.6%)	36 (5.9%)	5 (2.4%)	38 (5.6%)	*1-4
**MACE Type (Mutually Exclusive), n (%)[a,b]**							
(1) Multiple Types	326 (39.8%)	217 (39.6%)	109 (40.1%)	236 (38.7%)	90 (42.9%)	266 (39.1%)	60 (43.2%)
(2) Myocardial Infarction	148 (18.0%)	99 (18.1%)	49 (18.0%)	113 (18.5%)	35 (16.7%)	126 (18.5%)	22 (15.8%)
(3) Ischemic Stroke	77 (9.4%)	58 (10.6%)	19 (7.0%)	63 (10.3%)	14 (6.7%)	69 (10.1%)	8 (5.8%)
(4) Coronary Revascularization	230 (28.0%)	142 (25.9%)	88 (32.4%)	164 (26.9%)	66 (31.4%)	184 (27.0%)	46 (33.1%)
(5) Cardiovascular Death	39 (4.8%)	32 (5.8%)	7 (2.6%)	34 (5.6%)	5 (2.4%)	36 (5.3%)	*1-4

Abbreviations: Lp(a): lipoprotein(a); MACE: major adverse cardiovascular event

[a] Myocardial infarction and ischemic stroke include non-fatal events only.

[b] For MACE type with mutually exclusive categories, percents sum to 100 within the column.

Supplemental Table S11 Kaplan-Meier Time-to-First MACE Results

**Metric**	**Lp(a) ≤50 mg/dL**	**Lp(a) >50 mg/dL**	**Lp(a) >70 mg/dL**	**Lp(a) >90 mg/dL**
Summary of Follow-up	N = 5,163	N = 1,728	N = 1,254	N = 828
Patients with MACE - number (%)	548 (10.6%)	272 (15.7%)	210 (16.7%)	139 (16.8%)
Censored Patients - number (%)	4,615 (89.4%)	1,456 (84.3%)	1,044 (83.3%)	689 (83.2%)
MACE-Free KM Probability (95% CI)				
0 Months	97.5% (97.1%, 97.9%)	95.9% (95.0%, 96.9%)	95.4% (94.2%, 96.5%)	95.2% (93.7%, 96.6%)
6 Months	95.3% (94.8%, 95.9%)	91.4% (90.1%, 92.7%)	91.0% (89.4%, 92.6%)	90.7% (88.7%, 92.7%)
12 Months	94.6% (93.9%, 95.2%)	90.0% (88.6%, 91.5%)	89.7% (88.0%, 91.4%)	89.5% (87.3%, 91.6%)
18 Months	93.4% (92.7%, 94.1%)	89.1% (87.5%, 90.6%)	88.4% (86.5%, 90.3%)	88.1% (85.8%, 90.5%)
24 Months	92.0% (91.2%, 92.9%)	87.4% (85.7%, 89.1%)	86.5% (84.4%, 88.6%)	86.6% (84.0%, 89.2%)
30 Months	90.6% (89.7%, 91.5%)	86.1% (84.2%, 88.0%)	85.0% (82.6%, 87.3%)	85.0% (82.1%, 87.9%)
36 Months	89.5% (88.4%, 90.5%)	84.7% (82.7%, 86.8%)	83.2% (80.6%, 85.8%)	83.2% (80.0%, 86.5%)
42 Months	88.4% (87.3%, 89.5%)	83.2% (81.0%, 85.5%)	81.5% (78.7%, 84.3%)	81.2% (77.6%, 84.8%)
48 Months	87.4% (86.2%, 88.6%)	82.3% (80.0%, 84.7%)	80.4% (77.4%, 83.4%)	80.8% (77.1%, 84.5%)
54 Months	86.2% (84.9%, 87.5%)	79.8% (77.1%, 82.5%)	77.4% (74.0%, 80.8%)	75.8% (71.3%, 80.4%)
60 Months	85.1% (83.7%, 86.5%)	77.4% (74.4%, 80.4%)	74.9% (71.2%, 78.7%)	73.5% (68.5%, 78.5%)
66 Months	83.3% (81.7%, 84.9%)	76.5% (73.4%, 79.7%)	74.5% (70.7%, 78.3%)	73.5% (68.5%, 78.5%)
72 Months	81.6% (79.8%, 83.4%)	72.9% (69.3%, 76.5%)	70.6% (66.2%, 75.1%)	70.3% (64.6%, 75.9%)
78 Months	79.2% (77.2%, 81.2%)	72.1% (68.4%, 75.9%)	69.5% (64.8%, 74.2%)	68.4% (62.2%, 74.5%)

Metric	Lp(a) ≤50 mg/dL	Lp(a) >50 mg/dL	Lp(a) >70 mg/dL	Lp(a) >90 mg/dL
84 Months	77.8% (75.5%, 80.0%)	68.8% (64.3%, 73.2%)	65.2% (59.5%, 70.9%)	62.8% (55.1%, 70.5%)
90 Months	76.9% (74.5%, 79.3%)	64.7% (58.9%, 70.4%)	60.7% (53.4%, 68.0%)	57.1% (46.8%, 67.3%)
96 Months	76.3% (73.7%, 79.0%)	64.7% (58.9%, 70.4%)	60.7% (53.4%, 68.0%)	57.1% (46.8%, 67.3%)

Abbreviations: CI: confidence interval; KM: Kaplan-Meier; Lp(a): lipoprotein(a); MACE: major adverse cardiovascular events

Supplemental Figure S1 Study Cohort Flowchart

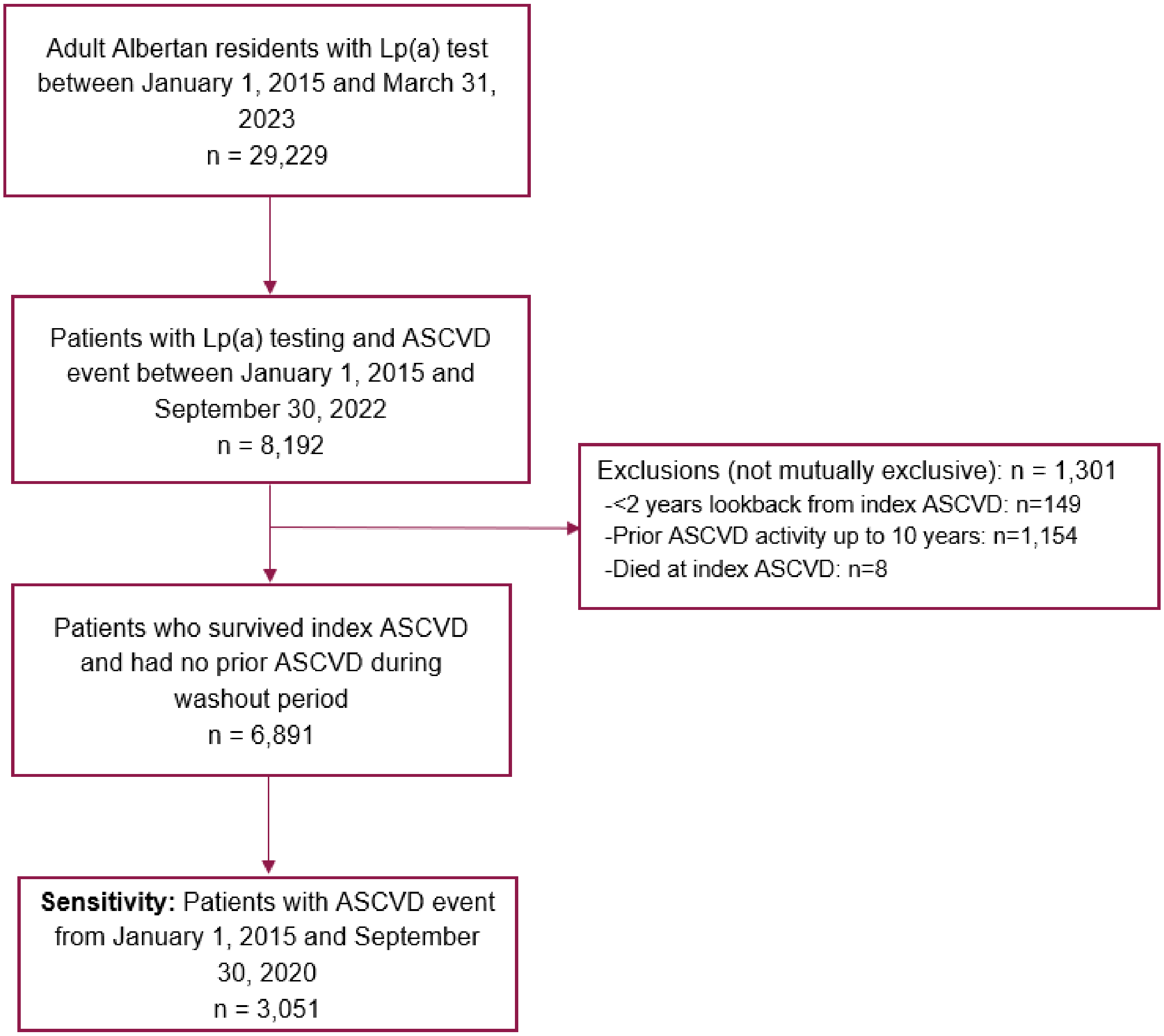

Abbreviations: ASCVD: atherosclerotic cardiovascular disease; Lp(a): lipoprotein(a)

Supplemental Figure S2 Kaplan-Meier Survival Probability by Lp(a) Thresholds

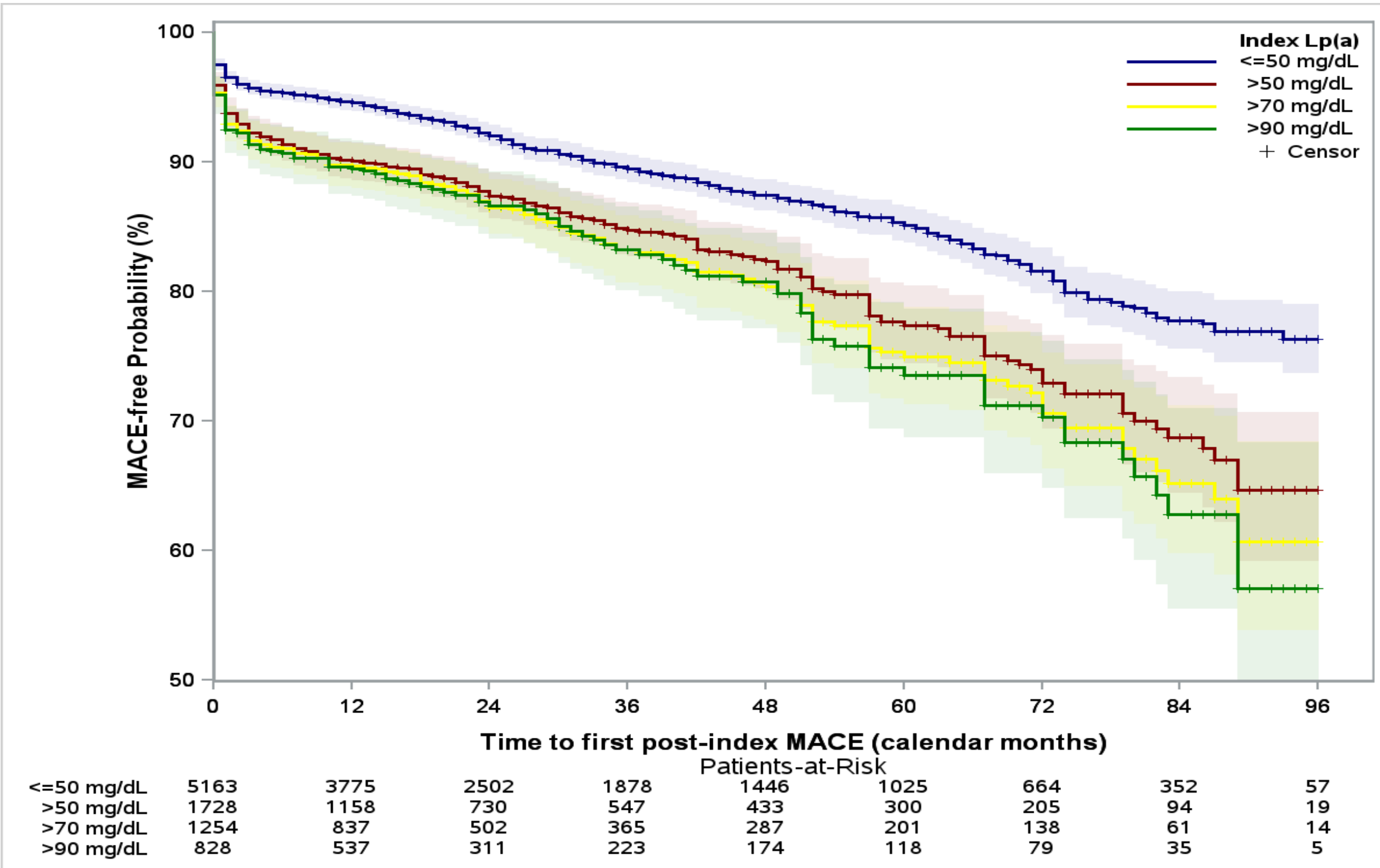

Abbreviations: Lp(a): lipoprotein(a); MACE: major adverse cardiovascular events
